# Vemurafenib improves muscle histopathology in a mouse model of *LAMA2*-related congenital muscular dystrophy

**DOI:** 10.1242/dmm.049916

**Published:** 2023-05-10

**Authors:** Ariany Oliveira-Santos, Marisela Dagda, Jennifer Wittmann, Robert Smalley, Dean J. Burkin

**Affiliations:** Department of Pharmacology, University of Nevada Reno, School of Medicine, Center for Molecular Medicine, Reno, NV 89557, USA

**Keywords:** *LAMA2*-CMD, Congenital muscular dystrophy, MDC1A, Laminin-α2, TGF-β, mTOR, Vemurafenib

## Abstract

Laminin-α2-related congenital muscular dystrophy (*LAMA2*-CMD) is a neuromuscular disease affecting around 1-9 in 1,000,000 children. *LAMA2*-CMD is caused by mutations in the *LAMA2* gene resulting in the loss of laminin-211/221 heterotrimers in skeletal muscle. *LAMA2*-CMD patients exhibit severe hypotonia and progressive muscle weakness. Currently, there is no effective treatment for *LAMA2*-CMD and patients die prematurely. The loss of laminin-α2 results in muscle degeneration, defective muscle repair and dysregulation of multiple signaling pathways. Signaling pathways that regulate muscle metabolism, survival and fibrosis have been shown to be dysregulated in *LAMA2*-CMD. As vemurafenib is a US Food and Drug Administration (FDA)-approved serine/threonine kinase inhibitor, we investigated whether vemurafenib could restore some of the serine/threonine kinase-related signaling pathways and prevent disease progression in the *dy^W−/−^* mouse model of *LAMA2*-CMD. Our results show that vemurafenib reduced muscle fibrosis, increased myofiber size and reduced the percentage of fibers with centrally located nuclei in *dy^W−/−^* mouse hindlimbs. These studies show that treatment with vemurafenib restored the TGF-β/SMAD3 and mTORC1/p70S6K signaling pathways in skeletal muscle. Together, our results indicate that vemurafenib partially improves histopathology but does not improve muscle function in a mouse model of *LAMA2*-CMD.

## Teaser

**Summary:** Vemurafenib partially improves muscle histopathology by restoring the TGF-β/SMAD3 and mTORC1/p70S6K signaling pathways but does not improve muscle function in a mouse model of *LAMA2*-related-congenital muscular dystrophy.

## INTRODUCTION

Laminin-α2-related congenital muscular dystrophy (*LAMA2*-CMD), also known as merosin-deficient congenital muscular dystrophy type 1A (MDC1A), is a severe form of congenital muscular dystrophy (CMD) that accounts for ∼30-40% of all CMDs in Europe (Allamand and Guicheney, 2002; Muntoni and Voit, 2004). The prevalence of *LAMA2*-CMD is not well known, but it is estimated to be about 1-9 per 1,000,000 of the total population (Durbeej, 2015; Graziano et al., 2015; Norwood et al., 2009).

*LAMA2*-CMD is caused by mutations in the *LAMA2* gene inherited in an autosomal recessive fashion that results in a complete loss or truncated expression of the laminin-α2 chain (Geranmayeh et al., 2010; Helbling-Leclerc et al., 1995). Laminin-α2 is essential for the assembly of laminin-211/221 heterotrimers, the major laminin isoforms expressed in adult skeletal muscle (Aumailley et al., 2005; Durbeej, 2010). Laminin-211 is the predominant isoform in the basement membrane surrounding the muscle fibers and axon–Schwann cell units of peripheral nerves, whereas laminin-221 is specifically found in the synaptic cleft of the neuromuscular junctions in adult skeletal muscles (Patton, 2000; Patton et al., 1997).

Laminin-α2 deficiency leads to the disruption of the basement membrane, resulting in muscle degeneration, fibrosis, inflammation (Gawlik and Durbeej, 2011, 2020; Pegoraro et al., 1996), abortive skeletal muscle regeneration (Kuang et al., 1999) and peripheral neuropathy (Mercuri et al., 1996; Shorer et al., 1995). *LAMA2*-CMD patients rarely achieve independent ambulation and exhibit severe hypotonia with muscle weakness and joint contractures at birth or within the first 6 months of life. Premature death is mainly caused by respiratory insufficiency in *LAMA2*-CMD patients (Geranmayeh et al., 2010; Jones et al., 2001; Philpot et al., 1995; Xiong et al., 2015; Zambon et al., 2020). Currently, there is no cure or effective treatment for *LAMA2*-CMD. The clinical practice guidelines recommend the multidisciplinary management of the symptoms. The standard medical care includes, among others, the treatment of joint contractures to promote joint and bone development, the use of orthotics and splinting for facilitation of standing and walking, activities to improve respiratory function, assisted ventilation, and management of pain (Oliveira et al., 2020; Wang et al., 2010). Clinical care only temporarily mitigates the symptoms of the disease.

Transgenic expression of the *Lama2* gene has been effective at preventing disease progression in mouse models of *LAMA2*-CMD (Kuang et al., 1998). However, laminin-α2 is encoded by a ∼10 kb transcript (Miyagoe-Suzuki et al., 2000), and therefore current gene therapy approaches that aim to replace the defective gene and restore laminin-α2 expression remain a challenge. Other gene therapy approaches aiming to stabilize the basement membrane of the muscle fibers have been shown to improve muscle disease in different mouse models of *LAMA2*-CMD (Aoki et al., 2013; Doe et al., 2011; Gawlik and Durbeej, 2010; Gawlik et al., 2004, 2018; Kemaladewi et al., 2017; McKee et al., 2009; Moll et al., 2001; Reinhard et al., 2017). However, the translation of gene therapies to the clinic remains a challenge at present owing to safety concerns including gene editing accuracy, insertional mutagenesis, limited long-term expression of the corrected gene and host immune response (Tang and Xu, 2020). Therefore, there is an urgent need to develop therapies that can slow disease progression and act on the downstream effects caused by the loss of laminin-α2, such as basement membrane disruption, muscle atrophy, apoptosis, regeneration, inflammation and fibrosis.

Several small molecules and biological treatments have been tested in laminin-α2-deficient mouse models – these include the administration of laminin-111 protein (Barraza-Flores et al., 2020; Rooney, 2012; van Ry et al., 2014) to compensate for the loss of laminin-211/221; IGF-1 (Accorsi et al., 2016; Lynch et al., 2001) and clenbuterol (Hayes and Williams, 1998) to promote muscle growth; omigapil (Erb et al., 2009; Yu et al., 2013), doxycycline (Girgenrath et al., 2009) and losartan (Elbaz et al., 2015) to inhibit apoptosis; anti-fibrotic (Elbaz et al., 2012; Meinen et al., 2012; Nevo et al., 2010, 2011) and anti-inflammatory (Connolly et al., 2002; Dadush et al., 2010) drugs; and several drugs targeting other important signaling pathways (Carmignac et al., 2011a,b; Fontes-Oliveira et al., 2018; Körner et al., 2014; Körner and Durbeej, 2016; Millay et al., 2008; Tomomura et al., 2011).

Studies show that loss of laminin-α2 leads to the dysregulation of several signaling pathways in the skeletal muscle of laminin-α2-deficient mouse models (de Oliveira et al., 2014; Durbeej, 2015; Nguyen et al., 2019; Taniguchi et al., 2006; Mehuron et al., 2014). The Ras-Raf-MEK-ERK signaling pathway, including proteins of the mitogen-activated protein kinase (MAPK) family, is an important signaling pathway that regulates several cellular processes, including metabolism, differentiation, proliferation, survival, apoptosis and inflammation (Peti and Page, 2013; Schultze et al., 2012; Turjanski et al., 2007), and its dysregulation has been associated with several human diseases (Kim and Choi, 2015). Enhanced extracellular signal-regulated kinase (ERK) activation is correlated to muscle wasting in cancer cachexia and Emery–Dreifuss muscular dystrophy, and its inhibition was shown to reduce skeletal muscle loss (Muchir et al., 2013; Penna et al., 2010). Increased ERK phosphorylation has also been reported in a *LAMA2*-CMD mouse model, and its inhibition after treatment with losartan improved forelimb and hindlimb grip strength and reduced fibrosis (Elbaz et al., 2012).

Vemurafenib, a US Food and Drug Administration (FDA)-approved serine/threonine kinase inhibitor (Zhang et al., 2017), is used for the treatment of melanomas (Czirbesz et al., 2019; Luke and Hodi, 2012) and reported to be effective in the treatment of Langerhans cell histiocytosis (Evseev et al., 2021) and gliomas (Bautista et al., 2014) in pediatric patients through its inhibition of the serine/threonine-protein kinase B-Raf, an upstream activator of ERK (Macdonald et al., 1993; McCubrey et al., 2007). Vemurafenib has also been reported to inhibit JNK signaling (Vin et al., 2013), an important signaling pathway in skeletal muscle development (Xie et al., 2018) and wasting (Mulder et al., 2020), and when combined with other drugs for the treatment of melanomas, it can modulate other signaling pathways (Li et al., 2022). Considering that the loss of laminin-α2 dysregulates several signaling pathways in *LAMA2*-CMD mouse models (Meinen et al., 2012;de Oliveira et al., 2014; Elbaz et al., 2012), we investigated whether vemurafenib could prevent disease progression by modulating the activity of serine/threonine kinases in the *dy^W−/−^* mouse model of *LAMA2*-CMD, in which *Lama2* is disrupted. Our studies show that short-term treatment with vemurafenib partially improves histomorphology, reduces fibrosis and downregulates the TGF-β/SMAD3 and mTORC1 pathways, but not the Ras-Raf-MEK-ERK signaling pathway. Despite the improvements observed, vemurafenib did not improve skeletal muscle function in *dy^W−/−^* mice, suggesting that the use of vemurafenib alone might not be a promising treatment option to prevent muscle disease progression in patients with *LAMA2*-CMD.

## RESULTS

### Muscle histopathology is partially improved with vemurafenib treatment in *dy^W−/−^* mice

The skeletal muscle histopathology in *dy^W−/−^* mice is characterized by a significant reduction in muscle area and the number of PAX7-positive cells during fetal development (Nunes et al., 2017). After birth, reduced body weight, muscle cross-sectional area and number of fibers are observed along with increased apoptosis and embryonic myosin heavy chain (eMHC, or MYH3) expression (Mehuron et al., 2014).

**Fig. 1.**
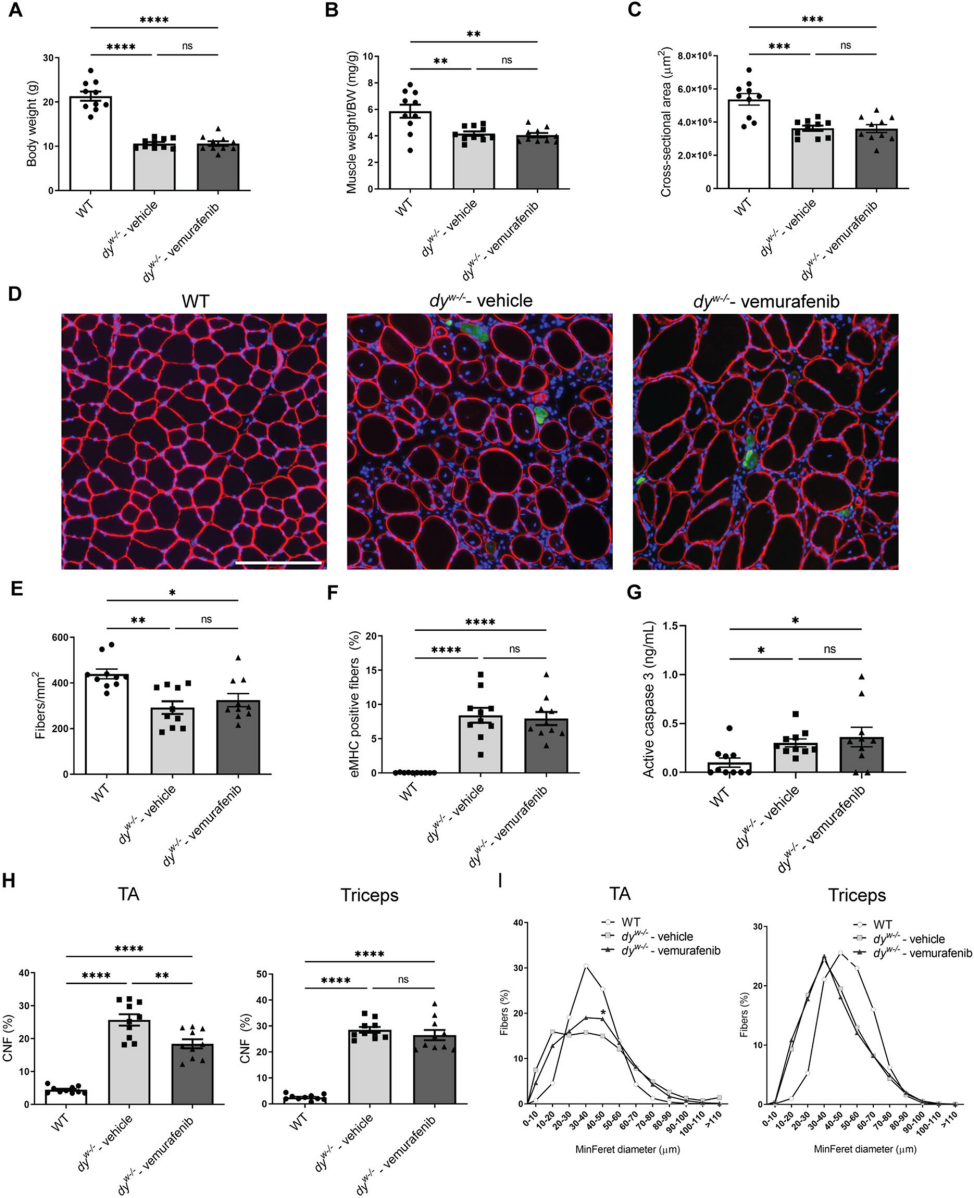
**Effects of vemurafenib on growth and histopathology of hindlimb muscles in the *dy^W−/−^* mouse model of *LAMA2*-CMD at 8 weeks of age.** (A) Body weight measurements of WT, *dy^W−/−^* vehicle-treated and *dy^W−/−^* vemurafenib-treated mice. (B) Quadriceps muscle weight/body weight (BW) ratio measurements. (C) Tibialis anterior (TA) cross-sectional area measurements. (D) Representative images showing dystrophin (red), embryonic myosin heavy chain (eMHC) (green) and nuclei (blue) staining in TA cryosections from WT mice (left), vehicle-treated *dy^W−/−^* mice (middle) and vemurafenib-treated *dy^W−/−^* mice (right). Scale bar: 200 µm. (E-I) Quantitative analysis of (E) number of fibers per mm^2^ of TA muscle, (F) percentage of eMHC-positive fibers in TA muscle, (G) active caspase 3 levels in the protein extract from gastrocnemius muscle, (H) percentage of fibers with centrally located nuclei (CNFs) in TA (left graph) and triceps (right graph), and (I) minimal Feret's (MinFeret) diameter distribution (percentage of the total number of fibers) in TA (left graph) and triceps (right graph) from WT mice, vehicle-treated *dy^W−/−^* mice and vemurafenib-treated *dy^W−/−^* mice. One-way ANOVA with uncorrected Fisher's LSD test was performed for the data that followed the normal distribution (body weight, muscle weight/body weight, cross-sectional area, fibers/mm^2^, eMHC-positive fibers, percentage of CNFs). Two-way ANOVA analysis was performed for MinFeret diameter distribution data (*P<0.05 denoting significance between vehicle-treated *dy^W−/−^* mice and vemurafenib-treated *dy^W−/−^* mice). Kruskal–Wallis test was performed for the data that did not follow the normal distribution (active caspase 3). All data are represented by statistical significance of mean±s.e.m. (*n*=10 for all groups). ns, not significant; *P<0.05; **P<0.01; ***P<0.001; ****P<0.0001.

In this study, we investigated whether treatment with 5 mg/kg vemurafenib could prevent further growth impairment from 3 weeks to 8 weeks of age in *dy^W−/−^* mice. As treatment was started at 3 weeks of age, this study examines the ability of vemurafenib to prevent muscle disease progression after onset. Vemurafenib treatment starting at 3 weeks of age did not show improvements in body weight (Fig. 1A), quadriceps weight (Fig. 1B), tibialis anterior (TA) cross-sectional area (Fig. 1C) and the number of fibers (Fig. 1E) in *dy^W−/−^* mice compared to vehicle control-treated *dy^W−/−^* mice. We verified that treatment with vemurafenib did not change the percentage of eMHC-positive fibers in the TA (Fig. 1D,F) and was not able to reduce active caspase 3 levels in the gastrocnemius (Fig. 1G) of 8-week-old *dy^W−/−^* mice. However, we observed that vemurafenib treatment significantly reduced the percentage of centrally nucleated fibers (CNFs) (Fig. 1D,H, left graph) and increased the percentage of muscle fibers with a minimal Feret's (MinFeret) diameter of 40-50 μm (Fig. 1D and I, left graph) in the TA of *dy^W−/−^* mice, compared to the percentages measured for vehicle control-treated mice. We also analyzed the percentage of CNFs and fiber diameter distribution in the triceps of *dy^W−/−^* mice, and we observed that the treatment with vemurafenib had no effect in reducing the percentage of CNFs and did not improve fiber diameter in the triceps of *dy^W−/−^* mice compared to those measured for vehicle control-treated mice (Fig. 1H,I, right graphs). Therefore, our data show that vemurafenib partially improves muscle histopathology by decreasing the percentage of CNFs and increasing the percentage of bigger fibers in the TA but not in the triceps of *dy^W−/−^* mice.

### Short-term treatment with vemurafenib reduces fibrosis and restores TGF-β1 and phosphorylated SMAD3 levels in *dy^W−/−^* mice

**Fig. 2.**
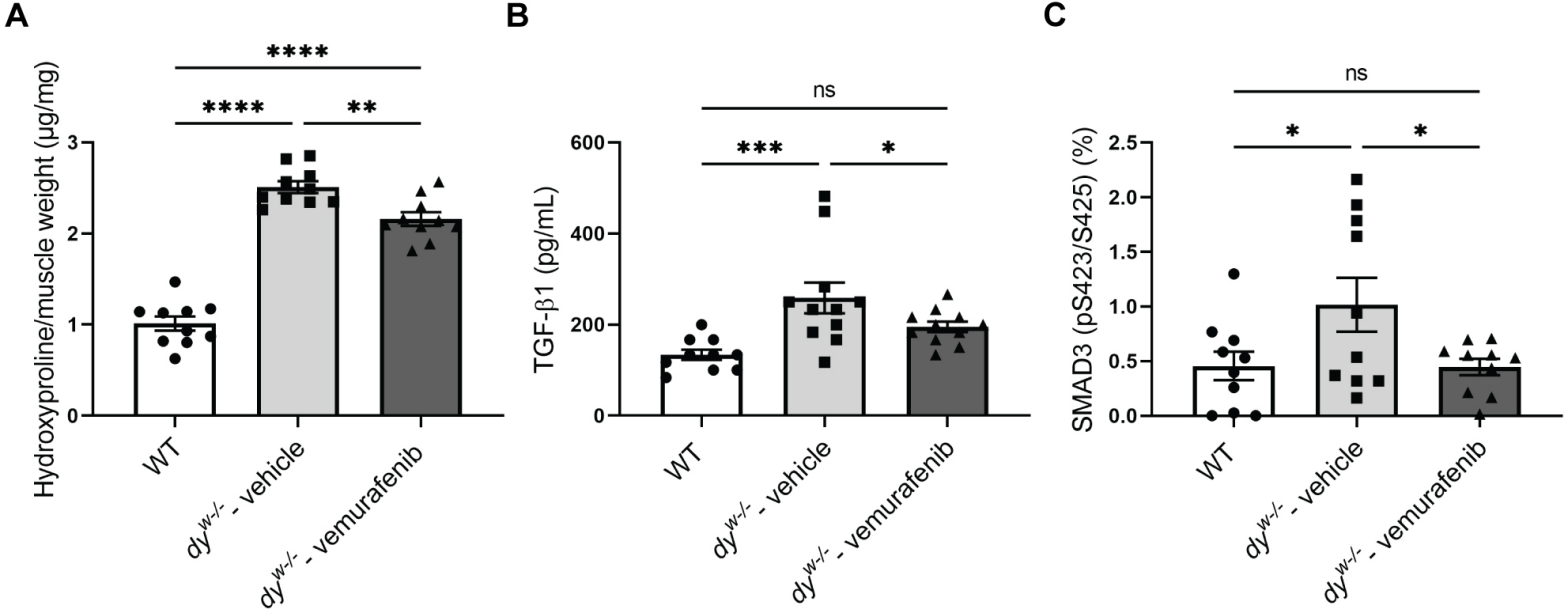
**Vemurafenib reduces fibrosis and restores the TGF-β/SMAD3 signaling pathway in *dy^W−/−^* mice.** Quantification of (A) hydroxyproline content in quadriceps muscle normalized by the muscle weight, (B) TGF-β1 levels and (C) percentage of SMAD3 (pS423/S425) in protein extracts of gastrocnemius muscle from WT mice, vehicle-treated *dy^W−/−^* mice and vemurafenib-treated *dy^W−/−^* mice. One-way ANOVA with uncorrected Fisher's LSD test represented by statistical significance of mean±s.e.m. (*n*=10 for all groups). ns, not significant; *P<0.05; **P<0.01; ***P<0.001; ****P<0.0001.

Interstitial fibrosis is considered an important feature in *LAMA2*-CMD muscles and it has been discussed to be a critical driver of the pathology (Accorsi et al., 2020; Taniguchi et al., 2006). Considering the importance of the fibrotic process in *LAMA2*-CMD pathology, we evaluated whether vemurafenib could inhibit the TGF-β/SMAD3 signaling pathway and prevent the progression of fibrosis (Ismaeel et al., 2019) in the *dy^W−/−^* mouse model. We verified that treatment with vemurafenib effectively reduced the levels of hydroxyproline, the major component of the collagen protein, in the quadriceps (Fig. 2A) and restored the levels of TGF-β1 (Fig. 2B) and phosphorylated SMAD3 (pS423/S425) (Fig. 2C) in the gastrocnemius of *dy^W−/−^* mice compared to those measured for *dy^W−/−^* vehicle-treated animals. These results show that vemurafenib is effective in preventing the progression of fibrosis and restores the TGF-β/SMAD3 signaling pathway to wild-type (WT) levels in the analyzed muscles of *dy^W−/−^* mice.

### Vemurafenib does not reduce inflammation in *dy^W−/−^* mice

**Fig. 3.**
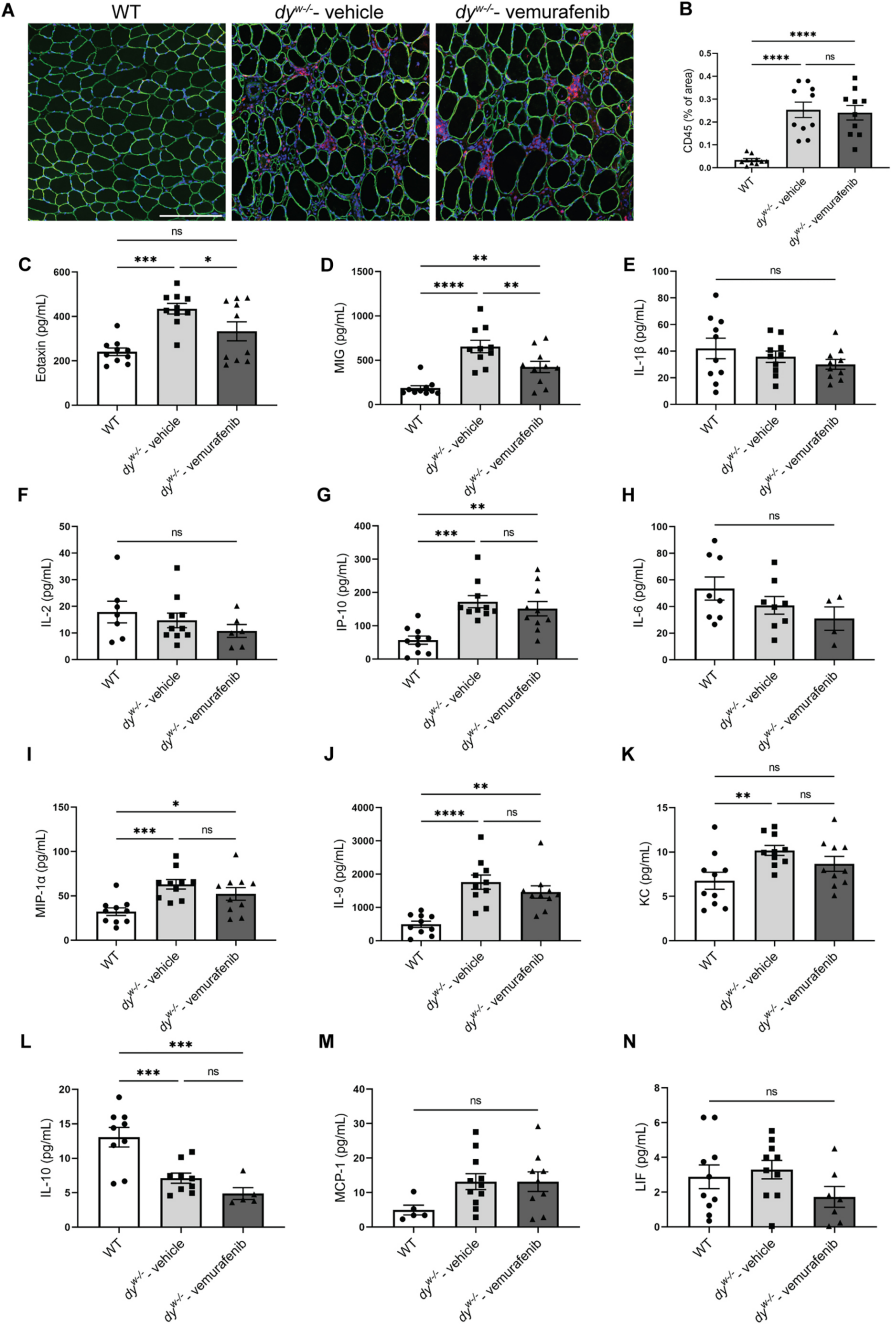
**Effects of vemurafenib on inflammatory cell infiltration and cytokine profile in hindlimbs from *dy^W−/−^* mice at 8 weeks of age.** (A) Representative images showing dystrophin (green), CD45-positive cells (red) and nuclei (blue) staining in TA cryosections from WT mice (left), vehicle-treated *dy^W−/−^* mice (middle) and vemurafenib-treated *dy^W−/−^* mice (right). Scale bar: 200 µm. (B-N) Quantification of (B) percentage of CD45-positive areas in TA muscle, and the levels of (C) eotaxin, (D) MIG, (E) IL-1β, (F) IL-2, (G) IP-10, (H) IL-6, (I) MIP-1α, (J) IL-9, (K) KC, (L) IL-10, (M) MCP-1 and (N) LIF in protein extracts of gastrocnemius muscle from WT mice, vehicle-treated *dy^W−/−^* mice and vemurafenib-treated *dy^W−/−^* mice. One-way ANOVA with uncorrected Fisher's LSD test was performed for the data that followed the normal distribution (CD45-positive area, MIG, IL-1β, IL-2, IL-6, MIP-1α, KC, MCP-1 and LIF measurements) and the Kruskal–Wallis test was performed for the data that did not follow the normal distribution (eotaxin, IP-10, IL-9 and IL-10 measurements). All data are represented by statistical significance of mean±s.e.m. Samples presenting cytokine levels below the Luminex detection limit were removed from the analysis. ns, not significant; *P<0.05; **P<0.01; ***P<0.001; ****P<0.0001. The individual symbols in the bar graphs represent the individual animals used in each experiment.

Inflammation is a hallmark of early disease progression in *LAMA2*-CMD patients (Pegoraro et al., 1996). Similarly, increased immune cell infiltration can be detected in *dy^W−/−^* mice as early as 1 week of age (Mehuron et al., 2014). In this context, we evaluated the effects of vemurafenib on the inflammatory response in *dy^W−/−^* mice. Our results corroborate with literature data demonstrating increased inflammatory cell infiltration in the *dy^W−/−^* skeletal muscle. However, no improvements in the inflammatory cell infiltration area were observed in the TA of *dy^W−/−^* mice after treatment with vemurafenib (Fig. 3A,B). We also detected increased levels of pro-inflammatory cytokines in the gastrocnemius muscle of 8-week-old *dy^W−/−^* mice, such as IP-10 (or CXCL10) (Fig. 3G), MIP-1α (CCL3) (Fig. 3I), IL-9 (Fig. 3J) and KC (CXCL1) (Fig. 3K), and reduced levels of the anti-inflammatory cytokine IL-10 (Fig. 3L). Vemurafenib treatment did not change the levels of these cytokines compared with their levels in *dy^W−/−^* vehicle-treated animals. Interestingly, the treatment with vemurafenib significantly restored the levels of eotaxin (CCL11) (Fig. 3C), an eosinophil chemoattractant (Rankin et al., 2000), and reduced the levels of monokine induced by γ interferon (MIG/CXCL9) (Fig. 3D), an important chemokine in inflammatory myopathies expressed by macrophages and T cells (Paparo, 2019). Luminex analysis showed no differences in the levels of IL-1β (Fig. 3E), IL-2 (Fig. 3F), IL-6 (Fig. 3H), MCP-1 (CCL2) (Fig. 3M) and LIF (Fig. 3N) in the three groups analyzed. Our data show that despite reducing the levels of two important chemokines, vemurafenib was not efficient in inhibiting inflammation in *dy^W−/−^* mice.

### Vemurafenib treatment reduced mTORC1 signaling pathway activation in *dy^W−/−^* mice

**Fig. 4.**
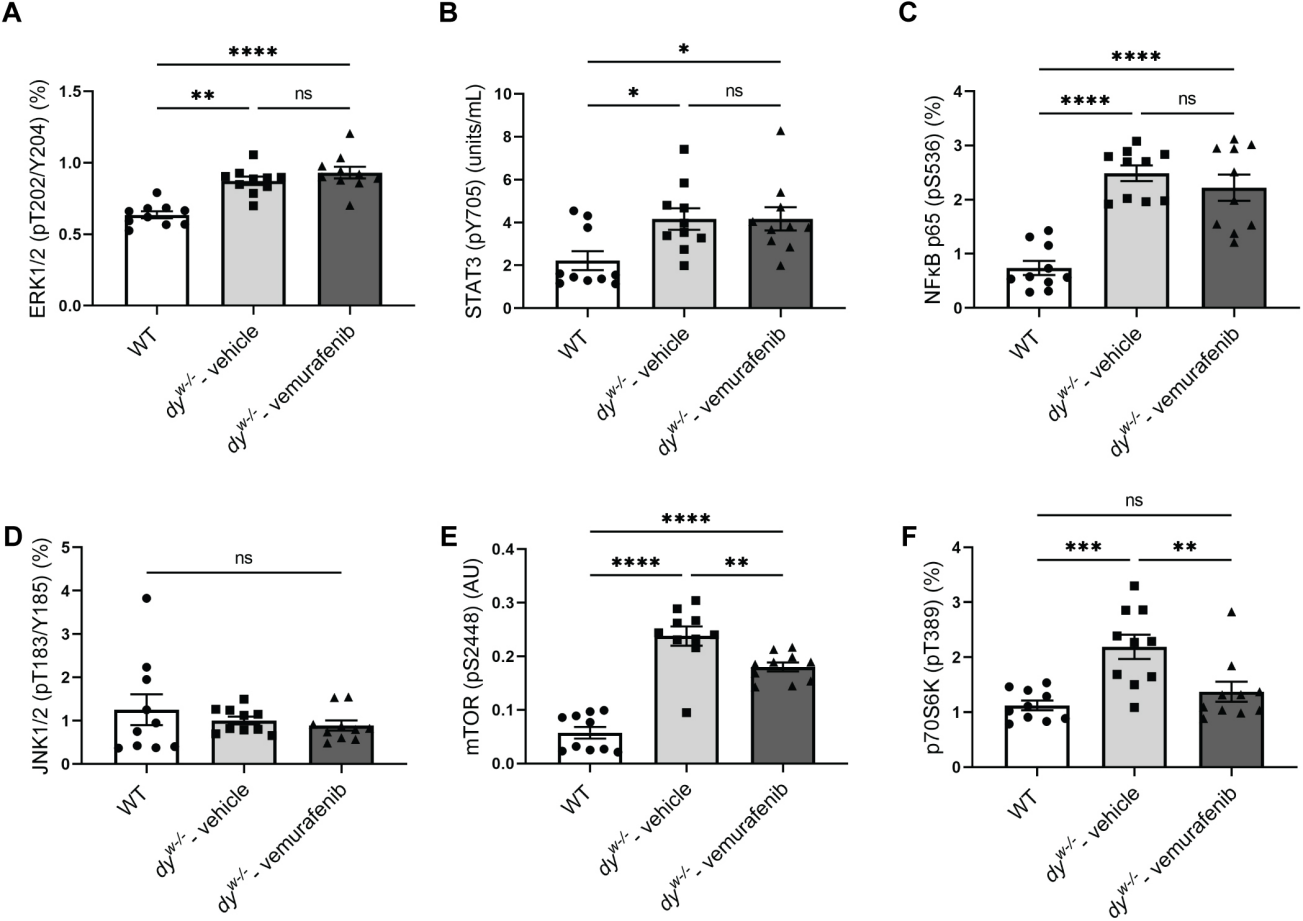
**Vemurafenib inhibited mTOR and p70S6K activation in *dy^W−/−^* mice.** Levels of (A) phosphorylated ERK1/2 (pT202/Y204), (B) phosphorylated STAT3 (pY705), (C) phosphorylated NFκB p65 (pS536), (D) phosphorylated JNK1/2 (pT183/Y185), (E) phosphorylated mTOR (pS2448) and (F) phosphorylated p70S6K (pT389) in protein extracts of gastrocnemius muscle from WT mice, vehicle-treated *dy^W−/−^* mice and vemurafenib-treated *dy^W−/−^* mice. One-way ANOVA with uncorrected Fisher's LSD test was performed for the data that followed the normal distribution [ERK1/2 (pT202/Y204), mTOR (pS2448) and NFκB p65 (pS536)]. Kruskal–Wallis test was performed for the data that did not follow the normal distribution [STAT3 (pY705), JNK1/2 (pT183/Y185) and p70S6K (pT389)]. All data are represented by statistical significance of mean±s.e.m. (*n*=10 for all groups). ns, not significant; *P<0.05; **P<0.01; ***P<0.001; ****P<0.0001.

Several muscle signaling pathways that regulate metabolism, inflammation and fibrosis have been described to be dysregulated in *LAMA2*-CMD patients and animal models, including ERK, NFκB and STAT3 (Carmignac et al., 2011a; de Oliveira et al., 2014; Durbeej, 2015; Elbaz et al., 2012, 2015; Nguyen et al., 2019; Nunes et al., 2017; Taniguchi et al., 2006; Mehuron et al., 2014). As vemurafenib is a MEK/ERK inhibitor, we first investigated whether the short-term treatment with vemurafenib would effectively modulate this pathway by inhibiting ERK phosphorylation. We verified that 5 mg/kg of vemurafenib did not inhibit ERK activation in gastrocnemius of 8-week-old *dy^W−/−^* mice (Fig. 4A). We next analyzed the effects of vemurafenib on STAT3 and NFκB activation, which have been described to be involved with inflammation, fibrosis, muscle atrophy and muscle wasting, and upregulated due to laminin-α2 deficiency (Chakraborty et al., 2017; Guadagnin et al., 2018; Ma et al., 2017; Sartori et al., 2021). Vemurafenib did not inhibit STAT3 (Fig. 4B) and NFκB p65 (RELA) activation (Fig. 4C) in *dy^W−/−^* mice.

As we observed an improvement in myofiber atrophy after treatment with vemurafenib (Fig. 1D,I), we analyzed the levels of phosphorylated JNK1/2, a MAPK family member, described to be involved in myofiber atrophy (Mulder et al., 2020). No difference was observed in the levels of phosphorylated JNK1/2 (Fig. 4D) in the groups analyzed.

Considering the importance of the serine/threonine kinase mTOR in metabolism regulation (Bodine et al., 2001; Fingar and Blenis, 2004; Giguère, 2018) and the well-described effects of sustained activation of the mTORC1 signaling pathway to promote skeletal muscle atrophy and loss in different pathological processes (Castets et al., 2013; Chiarini et al., 2019; Tang et al., 2014, 2019), we next investigated whether the increased myofiber diameter observed after treatment with vemurafenib was associated with the modulation of the mTORC1 signaling pathway in our animal model of *LAMA2*-CMD. We verified increased levels of phosphorylated mTOR (pS2448) (Fig. 4E) and phosphorylated p70S6K (RPS6KB1) (pT389), a downstream target of mTOR (Fig. 4F) in gastrocnemius of 8-week-old *dy^W−/−^* mice, and treatment with vemurafenib reduced the levels of phosphorylated mTOR (pS2448) (Fig. 4E) and restored the levels of phosphorylated p70S6K (Fig. 4F) to WT levels. Our results show that vemurafenib acted to normalize the mTORC1 signaling pathway in *dy^W−/−^* mice.

### Ubiquitin-proteasome-related pathways and autophagy are not modulated by vemurafenib in 8-week-old *dy^W−/−^* mice

**Fig. 5.**
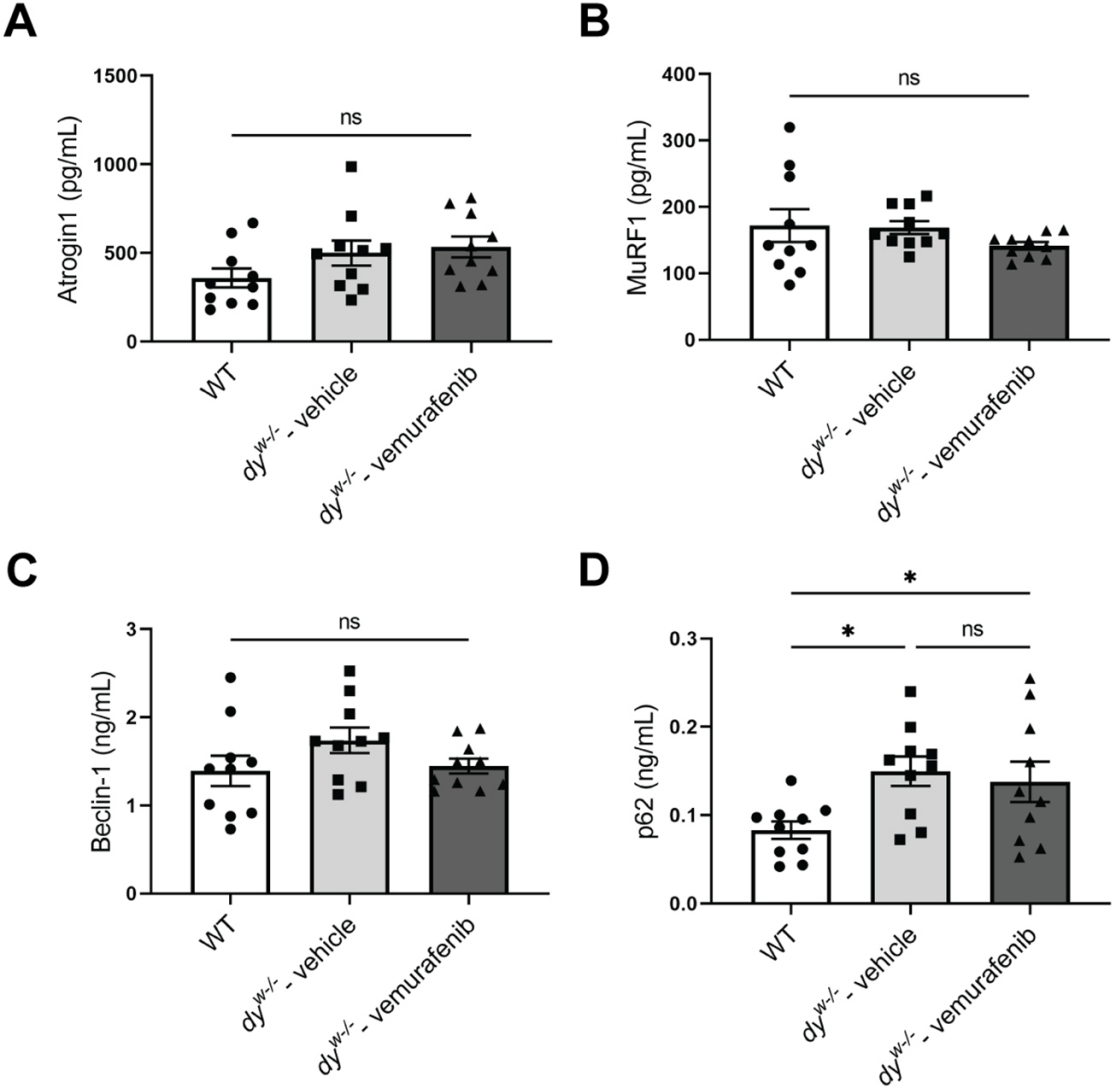
**The E3 ubiquitin ligases atrogin1 and MuRF1 are not upregulated in *dy^W−/−^* mice at 8 weeks of age.** Protein levels of (A) atrogin1, (B) MuRF1, (C) beclin-1 and (D) p62 in protein extracts of gastrocnemius muscle from WT mice, vehicle-treated *dy^W−/−^* mice and vemurafenib-treated *dy^W−/−^* mice. One-way ANOVA with uncorrected Fisher's LSD test represented by statistical significance of mean±s.e.m. (*n*=10 for all groups). ns, nonsignificant; *P<0.05.

Sustained activation of mTORC1 has been described to promote muscle atrophy by increasing the expression of E3 ubiquitin ligases and impairing autophagy (Castets et al., 2013). To verify whether the ubiquitin-proteasome system and autophagy are impaired and/or being modulated by vemurafenib in *dy^W−/−^* mice at 8 weeks of age, we quantified the protein levels of two important ubiquitin-proteasome-related components, atrogin1 (FBXO32) and MuRF1 (TRIM63) (Khalil, 2018), and two proteins involved in autophagy, beclin-1 and p62 (SQSTM1) (Glick et al., 2010). We did not observe significant differences in the protein levels of atrogin1 (Fig. 5A), MuRF1 (Fig. 5B) and beclin-1 (Fig. 5C) in 8-week-old WT, *dy^W−/−^* vehicle-treated and *dy^W−/−^* vemurafenib-treated mice. However, we detected increased levels of p62 (Fig. 5D) in *dy^W−/−^* vehicle-treated mice compared to WT mice, suggesting that autophagy might be impaired in *dy^W−/−^* mice at 8 weeks of age. Treatment with vemurafenib was not effective at reducing p62 in *dy^W−/−^* mice. Our results suggest that the ubiquitin-proteasome system and autophagy are not modulated by vemurafenib in *dy^W−/−^* mice at 8 weeks of age.

### Skeletal muscle function is not improved after vemurafenib treatment in *dy^W−/−^* mice

**Fig. 6.**
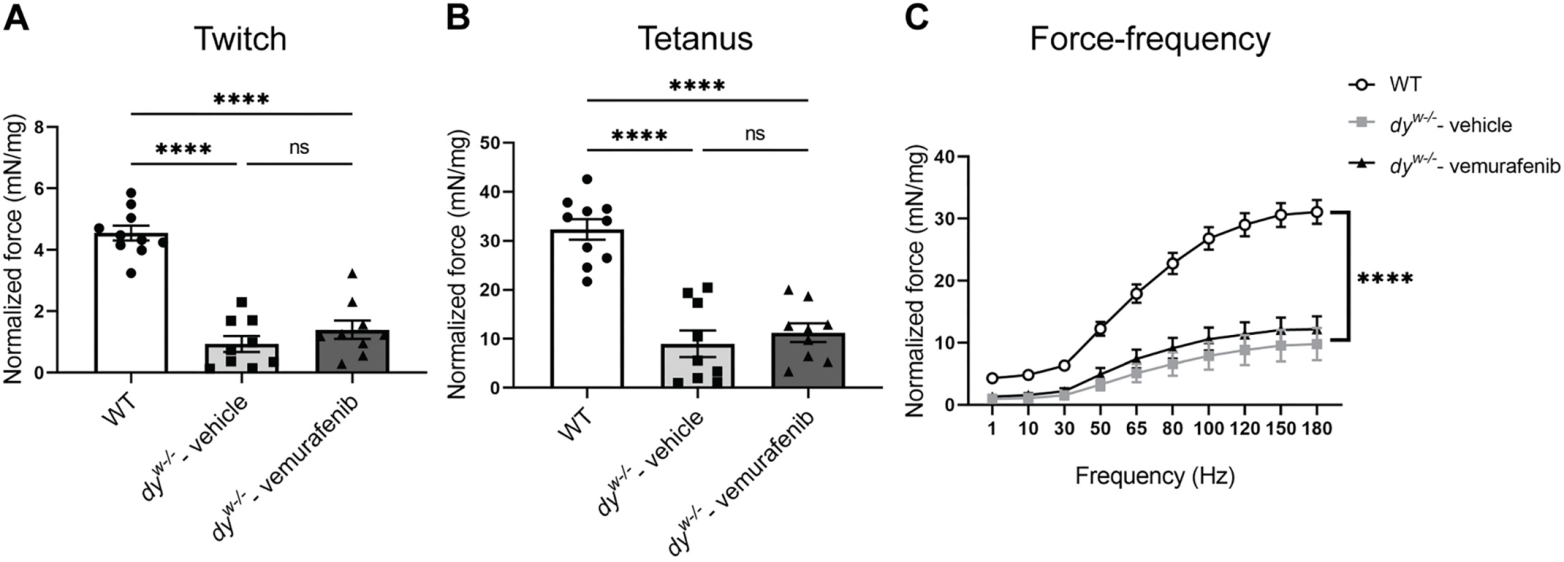
***Ex vivo* contractility analysis of EDL muscle in *dy^W−/−^* mice.** Normalized force measurements for (A) twitch, (B) tetanus and (C) force frequency. One-way ANOVA with uncorrected Fisher's LSD test was performed for the twitch and tetanus data and two-way ANOVA analysis was performed for the force-frequency data (****P<0.0001 denoting significance between WT and vehicle- or vemurafenib-treated *dy^W−/−^* mice). All data are represented by statistical significance of mean±s.e.m. (*n*=9 for all groups). ns, not significant; ****P<0.0001.

To investigate whether the improvements in histopathology and regulation of the mTORC1 signaling pathway were sufficient to improve skeletal muscle function in vemurafenib-treated *dy^W−/−^* mice, we performed the *ex vivo* contractility assay using the extensor digitorum longus (EDL) muscle. Our data show no significant improvements in strength in twitch (Fig. 6A), tetanus (Fig. 6B) and force-frequency measurements (Fig. 6C) in *dy^W−/−^* vemurafenib-treated mice compared to *dy^W−/−^* vehicle-treated mice. Our results indicate that treatment with 5 mg/kg vemurafenib from 3 to 8 weeks of age does not improve muscle strength in *dy^W−/−^* mice.

## DISCUSSION

*LAMA2*-CMD is a severe form of congenital muscular dystrophy characterized by hypotonia, progressive muscle weakness and respiratory insufficiency (Sarkozy et al., 2020). Only palliative care is available for *LAMA2*-CMD patients, which aims to reduce disease symptoms and improve quality of life (Wang et al., 2010). Although several treatment approaches have been shown to improve muscle histopathology in mouse models of *LAMA2*-CMD (Aoki et al., 2013; Durbeej, 2015; Hagiwara et al., 2006; Qiao et al., 2005), only one clinical trial has been performed for this congenital muscular dystrophy. Santhera Pharmaceuticals completed a phase 1 clinical trial with omigapil, an anti-apoptotic drug, which was demonstrated to be safe and well tolerated in children with *LAMA2*-CMD. Owing to the short duration of the study, data showing disease-related improvements are not available (NCT01805024). The lack of clinical trials reinforces the need for new treatments that could be fast-tracked for *LAMA2*-CMD. In this context, our group evaluated the efficacy of vemurafenib in preventing disease progression in *dy^W−/−^* mice. As an FDA-approved drug, vemurafenib, if proven to be efficacious, could be fast-tracked for the treatment of *LAMA2*-CMD.

Our data showed that vemurafenib improved skeletal muscle histomorphology by increasing the percentage of muscle fibers with a MinFeret diameter of 40-50 μm and by reducing the percentage of CNFs from 25% in *dy^W−/−^* vehicle-treated mice to 18% in the TA of *dy^W−/−^* vemurafenib-treated mice. The treatment with vemurafenib did not increase regenerative capacity or reduce apoptosis in skeletal muscle. Therefore, vemurafenib was not able to prevent the loss of muscle from the hindlimbs of *dy^W−/−^* mice.

We did not observe a correlation between the percentage of CNFs, the percentage of eMHC-positive fibers and apoptosis in our animal model after treatment with vemurafenib, suggesting that the reduction in CNFs in the TA observed is not due to the reduction in the degeneration-regeneration process. Indeed, the displacement of nuclei in skeletal muscle fibers is not restricted to the regeneration process; the central nucleation can also be caused by skeletal muscle denervation (Carraro and Kern, 2016; Daou et al., 2020). *LAMA2*-CMD patients and animal models present with impaired motor nerve conduction owing to reduced axon diameter and thickness of the axon myelin sheath, which could cause central nucleation of myofibers (Nakagawa et al., 2001; Patton, 2000; Previtali and Zambon, 2020; Quijano-Roy et al., 2004). In *dy^W−/−^* mice, secondary atrophy is observed in the hindlimbs due to paralysis, which is discussed to be associated with peripheral neuropathy (Miyagoe-Suzuki et al., 2000). The forelimbs, such as the triceps, are not affected by paralysis in this animal model of *LAMA2*-CMD (Meinen et al., 2012). As we only observed the reduction in the percentage of CNFs in the TA and not in the triceps of *dy^W−/−^* mice after treatment with vemurafenib, we suggest that the action of vemurafenib to reduce the percentage of CNFs might be related to the reduction of the neuropathy associated with the loss of the laminin-α2 protein in the hindlimbs. Further studies will be required to evaluate whether the impaired nerve conduction might be contributing to the nuclei displacement and whether vemurafenib could be acting to improve *LAMA2*-CMD neuropathy in the hindlimbs of *dy^W−/−^* mice.

Extracellular matrix remodeling resulting in fibrotic tissue deposition in skeletal muscles is a hallmark of muscular dystrophies (Serrano and Muñoz-Cánoves, 2017). In *LAMA2*-CMD, fibrotic tissue deposition plays a central role in disease progression (Accorsi et al., 2020). In this study, we showed that treatment with vemurafenib significantly reduced fibrosis and restored the TGF-β/SMAD3 signaling pathway to the basal levels.

We also analyzed the effects of vemurafenib on the inflammatory response, another important feature of *LAMA2*-CMD (Pegoraro et al., 1996). We did not observe an overall improvement in the inflammatory response after the treatment with vemurafenib. The reduced levels of the chemoattractants eotaxin and MIG were not sufficient to decrease the infiltration of immune cells in the muscle of *dy^W−/−^* vemurafenib-treated mice. However, as we focused the analysis only on the pan-leukocyte marker CD45, we cannot exclude possible effects of vemurafenib in the differentiation, activation and recruitment of specific leukocyte subpopulations.

We next evaluated the effects of vemurafenib on signaling pathways dysregulated in the context of laminin-α2 deficiency. Vemurafenib was able to restore the mTORC1 signaling pathway but did not inhibit ERK, NFκB and STAT3 activation in *dy^W−/−^* mice. It is important to mention that this study demonstrates the overactivation of the mTORC1/p70S6K signaling pathway in the gastrocnemius muscle of *dy^W−/−^* mice. The mTOR signaling pathway is well described to be involved in protein synthesis, muscle hypertrophy and growth (Bodine et al., 2001; Schiaffino et al., 2021). However, sustained activation of mTORC1 can lead to pulmonary fibrosis (Gui et al., 2015) and drive neuromuscular junction structural alterations, resulting in myofiber denervation (Ang et al., 2022), muscle atrophy, loss of muscle mass (Tang et al., 2014, 2019) and late-onset myopathy by increasing the expression of the E3 ubiquitin ligases atrogin1 and MuRF1 and impairing autophagy (Castets et al., 2013). Inhibition of mTORC1 by rapamycin improves muscle pathology in the fukutin-deficient mouse model of dystroglycanopathy (Foltz et al., 2016) and the *mdx* mouse model of Duchenne muscular dystrophy (Eghtesad et al., 2011).

Our results did not show a correlation between the upregulation of the mTORC1/p70S6K signaling pathway with the atrogin1 and MuRF1 protein levels detected in gastrocnemius of *dy^W−/−^* mice, suggesting that in our animal model at 8 weeks of age, the overactivation of the mTORC1/p70S6K pathway does not modulate the ubiquitin-proteasome system. Interestingly, studies have shown an increase in global protein ubiquitination and mRNA expression of atrogin1 and MuRF1 in the *dy^3k^/dy^3k^* mouse model of *LAMA2*-CMD (Carmignac et al., 2011a). *dy^3k^/dy^3k^* mice have a complete deficiency of laminin-α2 (Miyagoe et al., 1997), whereas *dy^W−/−^* mice produce a small amount of truncated laminin-α2 protein lacking the LN domain (Guo et al., 2003). Therefore, we suggest that the differences in the ubiquitin-proteasome activation observed could be related to the different levels of laminin-α2 chain expression in the two animal models, which results in different phenotypes, disease progression and lifespan (Gawlik and Durbeej, 2020) and will likely impact laminin-α2-related signaling pathways in the skeletal muscle. However, we did observe an increase in the levels of p62, indicating that autophagy might be impaired in *dy^W−/−^* mice. As we did not observe any difference in the levels of beclin-1, another protein involved in autophagy, further studies to evaluate other autophagic markers are essential to confirm whether autophagy is impaired in *dy^W−/−^* mice at 8 weeks of age.

Together, our results indicate that vemurafenib partially improves muscle histopathology by reducing the percentage of CNFs, increasing the percentage of muscle fibers with bigger diameter in the TA, reducing fibrosis, and restoring the TGF-β/SMAD3 and mTORC1/p70S6K signaling pathways to WT levels in hindlimbs of *dy^W−/−^* mice. Future studies will be necessary to understand the correlation of mTORC1 overactivation with the neuropathy observed in the hindlimbs of the *dy^W−/−^* mice, as chronic activation of mTORC1 could drive neuromuscular junction structural alterations and myofiber denervation (Ang et al., 2022).

As a pharmacological therapy that modulates intracellular signaling pathways involved in fibrosis and metabolism, vemurafenib is not capable of restoring the myomatrix and, consequently, does not improve muscle strength in *dy^W−/−^* mice. However, vemurafenib was able to partially improve muscle histopathology and restore the TGF-β/SMAD3 and mTORC1/p70S6K signaling pathways to WT levels. Therefore, combinatorial treatment with vemurafenib and therapeutics that restore the myomatrix and improve other pathological features in *LAMA2*-CMD (Erb et al., 2009; Yu et al., 2013; Barraza-Flores et al., 2020; Rooney, 2012; van Ry et al., 2014) might be a more effective therapeutic approach for *LAMA2*-CMD and should be investigated in future preclinical studies.

## MATERIALS AND METHODS

### Study design

To evaluate the benefits of vemurafenib as a therapeutic for *LAMA2*-CMD, we performed a short-term treatment with 5 mg/kg vemurafenib (MedChemExpress, HY-12057) in the *dy^w−/−^* mouse model of *LAMA2*-CMD from 3 to 8 weeks of age. The *dy^w−/−^* mouse model is a severe model of the disease presenting low levels of truncated laminin-α2 chain, which represents fairly well a group of patients with *LAMA2*-CMD, and it is considered a relevant model for *LAMA2*-CMD preclinical studies. The mice were treated four times a week in the morning via oral gavage with 5 mg/kg vemurafenib diluted in 10% DMSO, 40% PEG300 (MedChemExpress, HY-Y0873), 5% Tween-80 (MedChemExpress, HY-Y1891) and 45% saline, as per the manufacturer's recommendations for *in vivo* administration. The vehicle-treated mice were treated with an equal volume of vehicle solution (10% DMSO, 40% PEG300, 5% Tween-80 and 45% saline). After 5 weeks of treatment, we evaluated skeletal muscle histology, apoptosis, hydroxyproline content, inflammation, intracellular signaling pathways and skeletal muscle function. Animals were assigned randomly to experimental groups and analyses were performed blinded to treatment allocation. Different doses of vemurafenib were tested and the most efficient dose (5 mg/kg) to reduce hydroxyproline content (measurement of fibrosis) was chosen (Fig. S1). Initial analysis to verify gender effects was made on five males and five females for each group. No clear differences were observed (Fig. S2) and both genders were used for the final analysis. The minimal number of animals in each experiment was determined using power analysis (power=0.8, α=0.05 and r=0.7).

### Animals

Heterozygous *dy^W^* mice (Kuang et al., 1998) (gift from Eva Engvall via Paul Martin; The Ohio State University, Columbus, OH, USA) were crossed to obtain homozygous *dy^w−/−^* mutants. The animals were treated according to the rules and regulations specified in the approved protocol from the University of Nevada Reno Institutional Animal Care and Use Committee. At the end of the study, mice were sacrificed by CO_2_ asphyxiation followed by cervical dislocation under the American Veterinary Medical Association guidelines for euthanasia. All mice were maintained in a pathogen-free animal care facility with access to food and water *ad libitum*.

### Protein extraction

Gastrocnemius protein was extracted in RIPA buffer (50 mM Tris pH 7.4, 1% NP-40, 0.5% sodium deoxycholate, 0.1% SDS, 150 mM NaCl, 2 mM EDTA, 50 mM NaF) containing protease inhibitor cocktail, sodium fluoride (NaF) and sodium orthovanadate (Na_3_VO_4_) phosphatase inhibitors. Extracts were centrifuged at 14,000 ***g*** at 4°C for 5 min to obtain the supernatant. Protein quantification was performed using the Pierce BCA protein assay kit (Thermo Fisher Scientific, 23227) according to the manufacturer's recommendations.

### Enzyme-linked immunosorbent assay

Enzyme-linked immunosorbent assay (ELISA) kits were used to measure levels of active caspase 3 (MyBioSource, MBS7210856), TGF-β1 (Abcam, ab119557), phosphorylated SMAD3 (pS423/S425) (Abcam, 186038), phosphorylated mTOR (pS2448) (RayBiotech, PEL-mTOR-S2448), phosphorylated p70S6K (pT389) (Abcam, ab176651), phosphorylated ERK1/2 (pT202/Y204) (Abcam, ab176640), phosphorylated STAT3 (pY705) (Invitrogen, KHO0481), phosphorylated NFκB p65 (pS536) (Abcam, ab176647), phosphorylated JNK1/2 (pT183/Y185) (Abcam, ab176645), atrogin1 (LSBio, LS-F35338), MuRF1 (MyBioSource, MBS2502946), beclin-1 (LSBio, LS-F35824) and p62 (MyBioSource, MBS039475) in the gastrocnemius protein homogenate, according to the manufacturer's instructions.

### Luminex xMAP immunoassay

Luminex xMAP immunoassay was performed in the University of California, Los Angeles (UCLA) Immune Assessment Core to quantify cytokine levels in the gastrocnemius protein homogenate. Mouse magnetic cytokine/chemokine kits were purchased from EMD Millipore and used per the manufacturer's instructions. Briefly, 25 μl diluted (1:2) samples were mixed with 25 μl magnetic beads and allowed to incubate overnight at 4°C while shaking. After washing the plates twice with wash buffer in a Biotek ELx405 washer, 25 μl of biotinylated detection antibody was added and the samples were incubated for 1 h at room temperature. Then, 25 μl streptavidin-phycoerythrin conjugate was added to the reaction mixture and incubated for another 30 min at room temperature. Following two washes, the beads were resuspended in sheath fluid, and fluorescence was quantified using a Luminex 200TM instrument (Luminex Corporation, TX, USA). Samples presenting cytokine levels below the Luminex detection limit were removed from the analysis.

### Immunofluorescence

Freshly collected tibialis anterior (TA) and triceps brachii muscles were rinsed in PBS and placed into a 2:3 (v/v) optimum cutting temperature compound (Tissue-TEK OCT compound, Sakura Finetek, 4583) to 30% sucrose/PBS medium inside a cryomold and frozen in liquid nitrogen-cooled isopentane. Tissues were then cryosectioned at 10 μm thickness using a Leica CM1950 cryostat. Sections were fixed in cold 4% paraformaldehyde in PBS for 10 min and incubated overnight with the following primary antibodies: anti-eMHC [Developmental Studies Hybridoma Bank (DSHB), F1.652-s; 5 µg/ml], anti-dystrophin [DSHB, MANDRA1 (7A10); 5 µg/ml], anti-dystrophin (Abcam, ab15277; 4 µg/ml) and anti-CD45 (Abcam, ab10558; 50 µg/ml). After washing with PBS, muscle sections were incubated with secondary antibodies conjugated with Alexa Fluor 488 (Invitrogen, A11001; 2 µg/ml) and Alexa Fluor 546 (Invitrogen, A11035; 2 µg/ml) at room temperature for 45 min and mounted with Vectashield Antifade Mounting Medium with 4′,6-diamidino-2-phenylindole (DAPI) (Vector Laboratories, H-1200-10). When staining with monoclonal mouse antibodies, the Mouse-On-Mouse (MOM) kit (Vector Laboratories, FMK-2201) was used.

A series of three non-consecutive sections were acquired using the Keyence digital microscope (Keyence Corporation of America, IL USA) and the images were analyzed using Image J software (National Institutes of Health, USA). The measurements of the number of fibers, fiber MinFeret diameter, CNFs, eMHC-positive fibers and the CD45-positive area were performed on three stitched whole sections of the TA muscle and averaged for each animal.

### Hydroxyproline assay

Quadricep muscle was minced overnight in 2 ml of 6 M hydrochloric acid at 110°C and the resulting hydrolysate (10 µl) was mixed with 150 µl isopropanol. Hydroxyproline oxidation was then performed for 10 min at room temperature with the addition of 72 µl of 1.4% chloramine-T (Sigma-Aldrich, 402869) in citrate buffer (0.385 M sodium acetate trihydrate, 0.24 M citric acid, 1.2% acetic acid, 0.85 M sodium hydroxide). Ehrlich's reagent [1 ml; 7.5g of 4-(dimethylamino) benzaldehyde, 25 ml ethanol, 1688 μl sulfuric acid] was then added and incubated for 30 min at 55°C. The absorbance measurement was performed at 558 nm, as previously described (Heydemann et al., 2005).

### *Ex vivo* contractility assay

Extensor digitorum longus (EDL) muscles were dissected from deeply anesthetized mice with 2.5% isoflurane and mounted between two platinum electrodes, clamped at one tendon, and attached at the other tendon to a force transducer placed in an oxygenated bath containing a physiologic salt solution (PSS buffer, pH 7.6) at 30°C. Experiments were performed using the isolated muscle test system for mice (Aurora Scientific) as described previously (https://treat-nmd.org/wp-content/uploads/2016/08/cmd-DMD_M.1.2.002.pdf; Sperringer and Grange, 2016). Data were analyzed using DMA software (Aurora Scientific), and the force was normalized by the EDL muscle weight.

### Statistical analysis

All data are expressed as mean±standard error mean (s.e.m.). The means of all data that followed the normal distribution were analyzed using one-way ANOVA with uncorrected Fisher's least significant difference (LSD) test, and the data that did not follow the normal distribution were analyzed using the Kruskal–Wallis test. Twitch and tetanus data were analyzed using one-way ANOVA with uncorrected Fisher's LSD test, and force-frequency data were analyzed using two-way ANOVA. Statistical analyses were performed using GraphPad Prism 9 software. *P*-values, *n-*values and symbols are described in the figure legends. *P<0.05, **P<0.01, ***P<0.001, ****P<0.0001 were considered statistically significant.

## Supplementary Material

10.1242/dmm.049916_sup1 Supplementary information
