# Supplementary material for: Vemurafenib improves muscle histopathology in a mouse model of LAMA2-related congenital muscular dystrophy
Source: Dis Model Mech. 2023 May 10;16(6):dmm049916. doi: 10.1242/dmm.049916 (PMC10184677; doi:10.1242/dmm.049916)
Supplement: Supplementary information [file dmm-16-049916-s1.pdf]

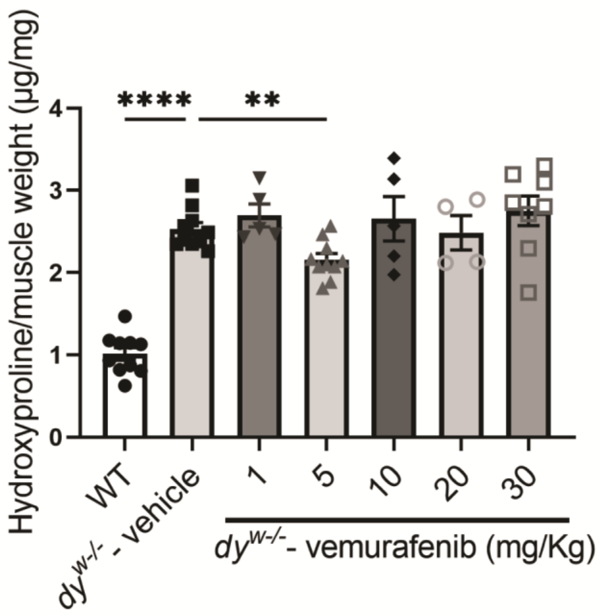

Fig. S1. Vemurafenib dose efficacy to reduce fibrosis in quadriceps muscle of *dy*$^{W–/–}$ mice. Quantification of hydroxyproline content (normalized by muscle weight) in quadriceps muscle of *dy*$^{W–/–}$ mice after treatment with 1, 5, 10, 20, and 30 mg/kg vemurafenib. One-way ANOVA analysis represented by statistical significance of mean ± SEM (WT, n=10; *dy*$^{W–/–}$- vehicle, n=10; *dy*$^{W–/–}$- vemurafenib 1mg/kg, n=5; *dy*$^{W–/–}$- vemurafenib 5 mg/kg, n=10; *dy*$^{W–/–}$- vemurafenib 10 mg/kg, n=5; *dy*$^{W–/–}$- vemurafenib 20 mg/kg, n=4; *dy*$^{W–/–}$- vemurafenib 30 mg/kg, n=8). The comparisons between *dy*$^{W–/–}$- vehicle and *dy*$^{W–/–}$- vemurafenib 1, 10, 20, and 30 mg/kg were not statistically significant.

Disease Models & Mechanisms • Supplementary information

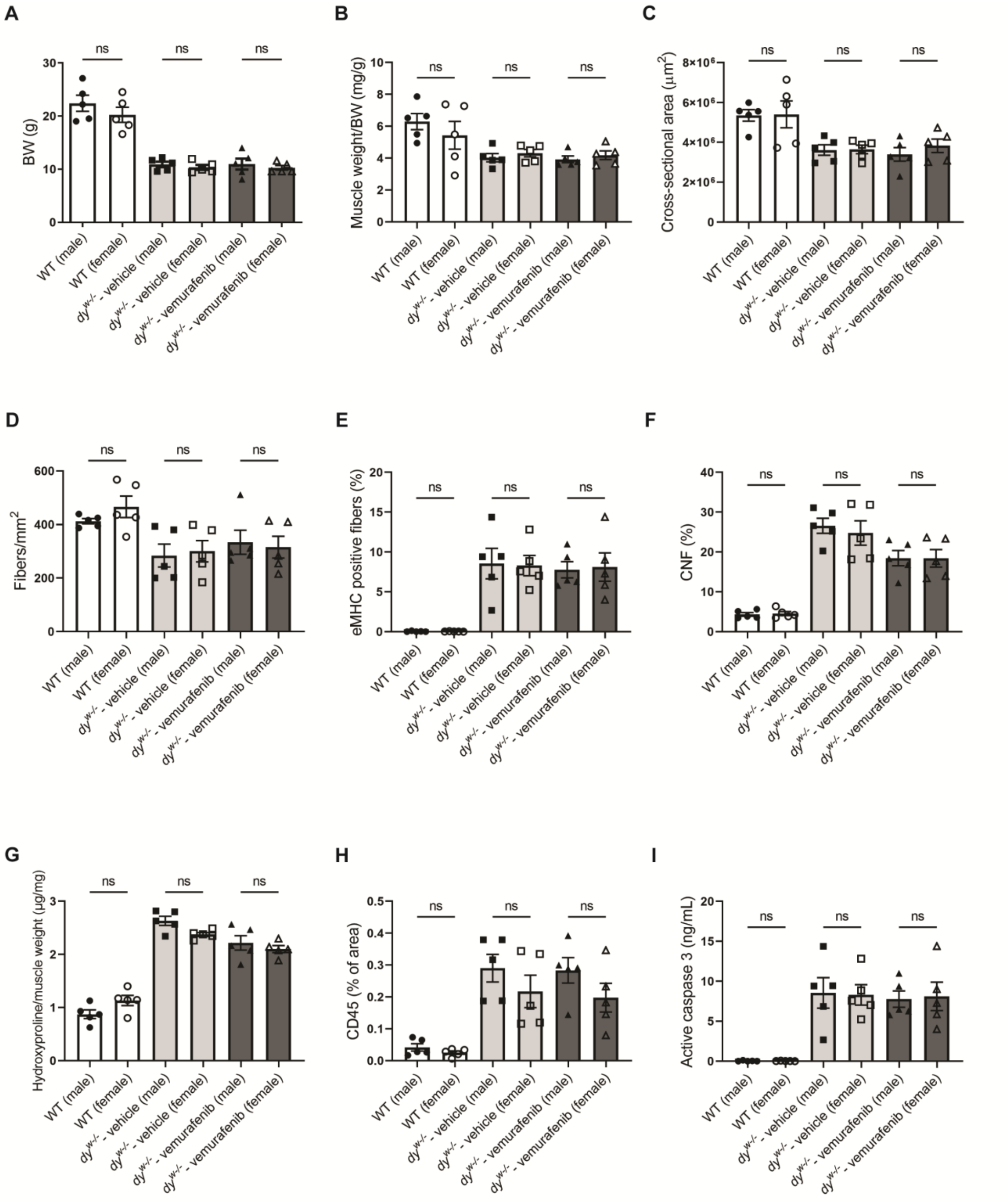

Fig. S2. Gender effects analysis in the *dy*$^{W-/-}$ mouse model of *LAMA2*-CMD.
Analysis of (**A**) body weight, (**B**) quadriceps muscle weight/body weight ratio, (**C**) tibialis anterior (TA) cross-sectional area, (**D**) number of fibers per mm^2 of TA muscle, (**E**)

percentage of embryonic myosin heavy chain (eMHC) positive fibers in TA muscle, (**F**) percentage of fibers with centrally located nuclei (CNF) in TA muscle, (**G**) hydroxyproline content (normalized by muscle weight) in the quadriceps muscle, (**H**) percentage of CD45 positive area in TA muscle, and (**I**) active caspase 3 levels in the protein extract from gastrocnemius muscle. One-way ANOVA analysis represented by statistical significance of mean ± SEM (n=5 for all groups). Only the comparisons between genders in the same group are indicated.
